# Optimization of a Method to Detect Autoantigen-Specific T-Cell Responses in Type 1 Diabetes

**DOI:** 10.3389/fimmu.2020.587469

**Published:** 2020-12-07

**Authors:** Yassmin Musthaffa, Hendrik J. Nel, Nishta Ramnoruth, Swati Patel, Emma E. Hamilton-Williams, Mark Harris, Ranjeny Thomas

**Affiliations:** ^1^Department of Endocrinology and Diabetes, Queensland Children’s Hospital, South Brisbane, QLD, Australia; ^2^The University of Queensland Diamantina Institute, The University of Queensland, Brisbane, QLD, Australia

**Keywords:** type 1 diabetes, proliferation, proinsulin, CD4+ T-cells, 5,6-carboxylfluorescein diacetate succinimidyl ester

## Abstract

The development of tolerizing therapies aiming to inactivate autoreactive effector T-cells is a promising therapeutic approach to control undesired autoimmune responses in human diseases such as Type 1 Diabetes (T1D). A critical issue is a lack of sensitive and reproducible methods to analyze antigen-specific T-cell responses, despite various attempts. We refined a proliferation assay using the fluorescent dye 5,6-carboxylfluorescein diacetate succinimidyl ester (CFSE) to detect responding T-cells, highlighting the fundamental issues to be taken into consideration to monitor antigen-specific responses in patients with T1D. The critical elements that maximize detection of antigen-specific responses in T1D are reduction of blood storage time, standardization of gating parameters, titration of CFSE concentration, selecting the optimal CFSE staining duration and the duration of T-cell stimulation, and freezing in medium containing human serum. Optimization of these elements enables robust, reproducible application to longitudinal cohort studies or clinical trial samples in which antigen-specific T-cell responses are relevant, and adaptation to other autoimmune diseases.

## Introduction

Type 1 Diabetes (T1D) is a chronic, incurable autoimmune disorder in which insulin-producing β-cells are selectively destroyed by islet-infiltrating T-cells (1, 2). The diagnosis of T1D is currently made at the onset of clinical symptoms. However, a long prodrome of autoimmune T and B cell activation leading to immune-mediated β-cell destruction precedes the onset of clinical diabetes. CD4^+^ T-cell responses are central to the pathogenesis of T1D (3–5) and several pathogenic self-epitopes have been reported. Human islet-infiltrating CD4^+^ T-cell clones were shown to recognize epitopes from proinsulin-derived C-peptide, as well as neoantigens such as hybrid insulin peptides (HIPs) (1, 5, 6). An important goal is the development of immunotherapies that prevent progression of β-cell destruction, thus intercepting T1D. Detecting and analyzing the function of islet-specific T-cells in humans has been challenging because of the low frequency of antigen-specific T-cells in the circulation and incomplete knowledge of their antigen specificity (7, 8). Consequently, there is currently an unmet need for a robust assay capable of tracking islet antigen-specific autoreactive T-cells to monitor immune-mediated activity while patients are undergoing immunotherapy. The desirable requirements of such an assay include 1) reliable performance with small blood volumes, 2) simplicity, 3) high sensitivity and specificity for detecting antigen specific T-cells in patients with or at-risk of T1D, 4) reproducibility, and 5) applicability to frozen peripheral blood mononuclear cells (PBMCs) (9).

Fluorescent dye-based proliferation assays represent a popular method to monitor antigen-specific T-cell responses *in vitro* (10, 11). In these assays, PBMCs are labeled with a fluorescent dye, such as carboxyfluorescein diacetate succinimidyl ester (CFSE), and divide in response to antigenic stimuli. The resulting progeny retain half the number of CFSE molecules of its parent. The corresponding decrease in fluorescence intensity can be measured by flow cytometry and identifies proliferating cells. Dividing cells can also be phenotypically characterized using antibodies specific for surface markers and/or intracellular cytokines. CFSE is an ideal dye to measure cell division in view of its capacity to label lymphocyte populations with a high fluorescent intensity. It is also compatible with a broad range of other fluorochromes, thus allowing multi-color flow cytometry, and single-cell sorting to clone antigen-reactive T-cells (12). The primary limitations of flow cytometric dye-dilution assays relate to reproducibility, resulting from variable background proliferation in unstimulated wells, subjective gating, and inter-operator variability of up to 78% (13). Background proliferation decreases assay sensitivity, particularly with antigens that induce low levels of T cell proliferation, e.g., due to low precursor frequency in circulation or low affinity T cell receptors (TCRs). Autoreactive T-cells may also be more sensitive to apoptosis than other antigen-specific T cells (14). For example, low affinity interactions between a pre-proinsulin peptide and the Human Leucocyte antigen (HLA)-A2 molecule reduces binding affinity of the TCR-peptide-HLA complex, potentially enabling T-cells to escape thymic deletion and enter the periphery (15).

Here we optimized the sensitivity of a published CFSE-based T-cell proliferation assay that demonstrated proinsulin_33-63_–specific CD4^+^ T cells in patients with recent-onset T1D (16). We used PBMCs from children with T1D for less than 3 months, to systematically optimize assay parameters that contribute to either death of the responding antigen-specific T-cells or that increase background proliferation in the absence of added antigen. The described protocol results in a reproducible assay that can be replicated in other laboratories and adapted for monitoring in other autoimmune diseases.

## Materials and Methods

### Subjects

Fresh blood (5–15 mls) was obtained from children (aged 2–16 years) with T1D duration of ≤ 3 months after informed consent. Recruited individuals carried alleles associated with high risk of T1D (HLA DR3-DQ2, DR4-DQ8, or DR3-DQ2/DR4-DQ8) as previously described (16). The study was approved by the Children’s Health Queensland, Mater Hospital, and University of Queensland Human Research Ethics Committees. T1D was defined according to the criteria from the American Diabetes Association (17).

### Cell Preparation

PBMCs were isolated by Ficoll Density (GE Healthcare, Sweden) gradient centrifugation then washed twice in phosphate buffered saline. Islet antigen-specific T-cell responses were measured using freshly isolated or thawed frozen PBMCs labeled with CFSE and cultured with or without islet peptides. Cells were cultured in complete medium [RPMI1640 medium, 1 U/ml penicillin/streptomycin/glutamate (Invitrogen, Thermo Fisher Scientific), 1 mM sodium pyruvate (Gibco, Thermo Fisher Scientific)] supplemented with 5% human serum (HS, Merck). Culture medium was filtered through a 0.2 µM filter (Sartorius-minisart) prior to culture or washing steps. PBMCs from single patients were analyzed separately.

### Cell Freezing

PBMCs were frozen at 10–25 × 10^6^ cells/vial in 1.0 ml of either 10% DMSO/fetal bovine serum (FBS, heat inactivated, Life Technologies), 10% DMSO/HS or a commercial serum-free freezing medium (CTL Cryo™ ABC Media Kit, Immunospot, USA). Counted cells were initially resuspended in 0.5 ml of either HS or CTL solution C and an equal volume of either 20% DMSO/FBS, 20% DMSO/HS or CTL solution AB was added. The cell suspension was aliquoted into cryovials and placed in a “Mr Frosty” (Nalgene, Thermo Scientific, Denmark) containing iso-propanol, at −80°C for 24–48 h before being transferred to liquid nitrogen.

Cells were thawed in a 37°C water-bath for 30 s and then added slowly to pre-warmed 5% HS/complete media containing 12.5 μg/ml DNase I (Sigma). The cell suspension was centrifuged, and the supernatant discarded. Cells were then resuspended in pre-warmed 5% HS/complete media supplemented with 6.25 μg/ml DNase I and rested for 30 min at 37°C 5% CO_2_. The cell suspension was passed through a cell strainer to remove dead cell clumps, then centrifuged and resuspended in complete medium.

### Synthetic Antigens

Tetanus toxoid (AJ Vaccines) was used at a concentration of 10 Lf/ml. Ultra-LEAF purified anti-human CD3 (Biolegend clone OKT3) was used at 0.1 µg/ml. Proinsulin_33-63_ (PI_33-63_, Sequence: EAEDLQVGQVELGGGPGAGSLQPLALEGSLQ, GL Biochem), was initially reconstituted to 16.55 M in DMSO (Sigma Aldrich) before making a working stock in PBS, of 2.5–5.0 mM for use at a final concentration of 10 µM as previously reported to reliably induce CD4^+^ T cell proliferation (16).

### CFSE Staining and T-Cell Stimulation

We followed methods previously described for a CFSE-based T-cell proliferation assay (5, 10) to measure antigen-specific CD4^+^ T-cell proliferation to PI_33-63_. Briefly, a total of 10–20 × 10^6^ PBMCs were re-suspended at 1 × 10^6^ cells/ml in pre-warmed (37°C) 1XPBS before staining with CFSE dye (CellTrace™ CFSE Cell Proliferation Kit for flow cytometry, Invitrogen, Thermo Fisher Scientific). CFSE concentration and staining times are as indicated in the figure legends. The CFSE labeling reaction was terminated with pre-warmed, filtered complete media containing 2.5% HS. The cells were washed and then re-suspended at a concentration of 2 × 10^6^ PBMCs/ml in pre-warmed, filtered 5% HS/RPMI. CFSE-stained cells (200 µl per well) were cultured in 96-well round bottom plates (Costar) for the number of days indicated in the figure legends, in 37°C 5% CO_2_ with medium alone (negative control) or with PI_33-63_, or with positive controls, tetanus toxoid and/or anti-CD3. In most experiments, cells were cultured in triplicate for each condition. Unstained cells were included in all cultures and used as single color stained controls to determine compensation settings on the flow cytometer.

### Flow Cytometry Analysis

After culture, triplicate wells were pooled, washed in saline and stained on ice with the following antibodies: anti-human CD3-APC/Cy7 (Mouse IgG1, κ, clone UCHT1), anti-human CD4-PE/Cy7 Antibody (Mouse IgG2b, κ, clone OKT4) and anti-human CD8a-Pacific Blue™ Antibody (Mouse IgG1, κ, clone HIT8a) (all from Biolegend, San Diego, CA). Gating of the major T cell populations was set using Fluorescence minus one (FMO) controls. LIVE/DEAD™ Fixable Aqua Dead Cell Stain Kit (Invitrogen, Thermo Fisher Scientific) was used to exclude dead cells. Harvested cells were acquired on a Gallios flow cytometer (Beckman Coulter) and analyzed with Kaluza software (Version 2.1, Beckman Coulter). Optimal voltage settings were determined for each experiment based on unstained and single stained samples. Viable CD3^+^CD4^+^ lymphocytes were gated. We acquired at least 2 × 10^5^ lymphocytes in order to identify 5,000 CD4^+^ CFSE^(undivided)^ cell events. The results are presented as a cell division index (CDI), the ratio of CD4^+^ T-cells that proliferated in response to antigen, relative to cells that proliferated in absence of antigen (10, 16). The CDI was calculated based on a fixed number of 5,000 CD4^+^ CFSE^(undivided)^ cells using the following formula:

$$CDI=\frac{number\;of\;divided\;CD4+Tcells\;per\;5,000\;CD4\;+Tcells\;in\;CFSE(undivided)\;from\;"with\;antigen"\;group\;}{number\;of\;divided\;CD4+Tcells\;per\;5,000\;CD4\;+Tcells\;in\;CFSE(undivided)\;from\;"without\;antigen"\;group\;}$$

A CDI of ≥3 was considered to represent the threshold for the positive control responses (16). Data from CFSE-based T-cell proliferation assays in which the CDI for both positive control antigen(s) did not exceed 3.0 were excluded from the analysis.

### VPD-450 Staining

Freshly isolated PBMCs (10–20 × 10^6^) were labeled with BD Horizon™ Violet Proliferation Dye 450 (VPD 450) at 10 × 10^6^ cells/ml in pre-warmed PBS with the concentrations of VPD450 as indicated. After 15 min the reaction was terminated with pre warmed 5% HS/complete medium. After washing, PBMC were resuspended to 2 × 10^6^/ml in pre-warmed, filtered 5% HS/complete medium.

### Assay Qualification

To determine the precision and reproducibility of the CFSE-based T-cell proliferation assay, we produced a standard operating procedure (SOP) and measured CD4^+^ T-cell responses to PI_33-63_ and tetanus toxoid from cryopreserved PBMC of a single patient diagnosed with T1D ≤ 3 months. We qualified the assay as fit-for purpose for research environments and non-regulated laboratories per recent guidelines for flow cytometry assays (18). Two analysts conducted independent experiments from the same sample. In each assay, six wells were set up for each condition. After 7 days of incubation, two wells were pooled together and analyzed as a single sample, resulting in three replicates. CDI values were calculated either from each replicate or using mean value of replicates of unstimulated cells to calculate CDI of stimulated cells. We calculated mean, standard deviation (SD), and coefficient of variation (CV) to provide intra-assay precision (%CV of CDI values from replicates of each condition), repeatability (mean %CV from assays of independent analysts) and inter-assay reproducibility (combined %CV from assays of independent analysts).

## Statistical Analysis

Pair-wise comparison between categorical variables was conducted with the Chi-square test, or the Fisher’s exact test if one or more cells in the contingency table had an expected frequency of ≤5 using Graphpad Prism (Version 7, San Diego, CA, USA) and R Statistical Software (Version 3.5.3, Foundation for Statistical Computing, Vienna, Austria). The Kruskal-Wallis test was used for multiple comparisons, followed by post-hoc Dunn’s Test (1964) and p-values were adjusted with the Benjamini-Hochberg method. Comparisons between group data were made using the paired two-tailed t-test. Statistical significance was defined as p < 0.05.

## Results

### Standardization of Gating Parameters

**Figure 1:**
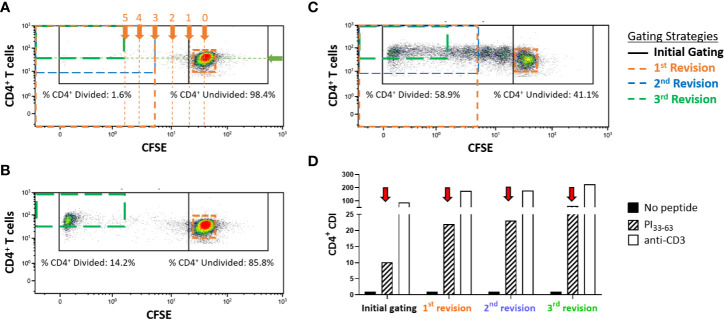
Representative flow cytometry plots displaying gating strategies to assess CD4^+^ T-cell proliferative responses in freshly isolated PBMC measured with the CFSE proliferation assay after a 7-day incubation. Cells were first gated on CD4^+^CD3^+^ live single cells. **(A)** PBMC that were incubated without antigen (no-peptide negative control), **(B)** PBMC incubated with PI_33-63_, **(C)** PBMC incubated with anti-CD3 (positive control). Proliferating cells are gated (left gates). In this example, 14.21% of CD4^+^ T-cells divided in response to incubation with PI_33-63_ when the 3^rd^ revised gating strategy was applied. Gates are set mathematically on the no-peptide condition and applied to all other conditions. The “initial gating” capturing the CFSE^(undivided)^ cells on the right, and CFSE^(divided)^ cells on the left is shown in black. The arrows labeled “0, 1, 2, 3, 4, 5” indicate the numbers of cell divisions, equivalent to MFI divided by 1, 2, 4, 8, 16, 32. The green arrow indicates the MFI of CD4, expressed by the CFSE^(undivided)^ population. **(D)** Revised gating strategies reduced capture of the no-peptide background proliferation and resulted in a higher CDI for all antigen-stimulated conditions. CDI, Cell division index.

To optimize a CFSE-based T-cell proliferation assay for detection of PI_33-63_ specific CD4^+^ T cells, CFSE-labeled freshly isolated PBMC from subjects with recent-onset T1D were stimulated for 7 days with or without PI_33-63_ or anti-CD3 as a positive control. We then standardized gating parameters in several stages, aiming to create an objective gating strategy, but reducing background proliferation in the no-peptide wells to a minimum. After setting a gate around the CFSE^(undivided)^ population to include all undivided cells, parameters for the borders of the CFSE^(divided)^ gate were calculated mathematically based on the Mean Fluorescence Intensity (MFI) of the CFSE^(undivided)^ population. The top and left border of the CFSE^(divided)^ gate was set at the top and left edge of the plot, respectively. The right-hand border of the CFSE^(divided)^ gate was calculated based on the fluorescence intensity of CFSE stained cells halving with each cell division. The right-hand border of the CFSE^(divided)^ gate was set at 3 cell divisions (MFI ^(undivided)^/2^3^) for the first and second revisions. The lower border of the CFSE^(divided)^ was raised to align with the lower border of the CFSE^(undivided)^ population. In order to capture antigen-specific proliferation while reducing the capture of background proliferation of no-peptide wells, the right-hand border of the CFSE^(divided)^ gate was set at 5 cell divisions (MFI ^(undivided)^/2^5^) and the bottom border position was objectively set at the MFI of the CD4^+^ staining of the CFSE^(undivided)^ population for the third revision (**Figures 1A–C**). Each gating revision decreased the number of events within the no-peptide divided cell gate and correspondingly increased the discrimination of the PI_33-63_ stimulated cells and CDI (**Figure 1D**).

### Titration of CFSE Concentration

**Figure 2:**
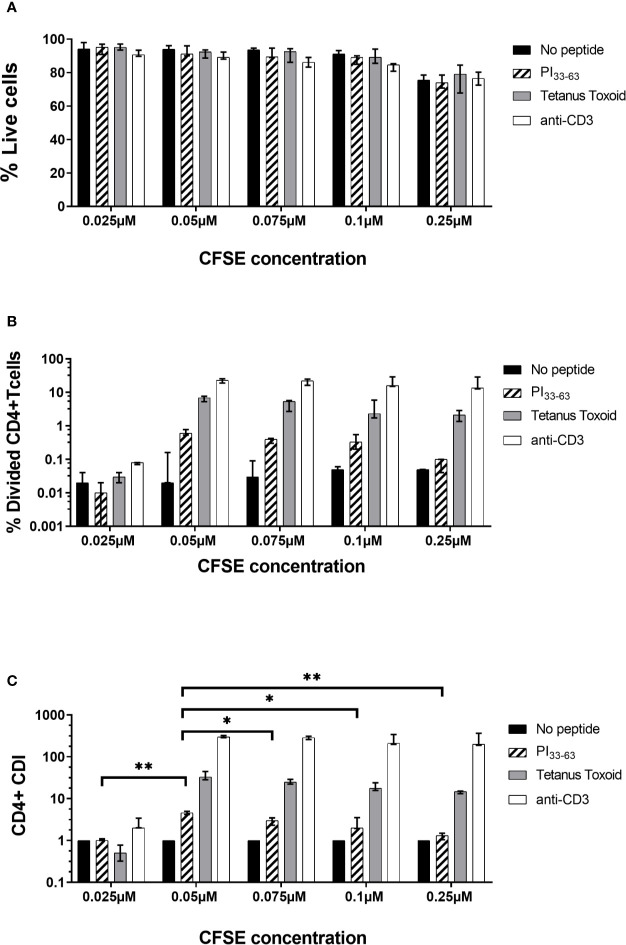
Titration of CFSE concentration. Freshly isolated PBMC from three patients were stained with different concentrations of CFSE as shown and incubated with PI_33-63_ (10 µM), tetanus toxoid (10 Lf/ml) or anti-CD3 antibodies (0.1 µg/ml) for 7 days. **(A)** Percentage of live cells after 7 days of incubation. **(B)** Percentage of Divided CD4^+^ T-cells. **(C)** CD4^+^ T-cell Division Index. Median value and range from three technical replicates are shown. CDI for each CFSE concentration was compared: *p < 0.05, **p < 0.01, paired t test.

CFSE is toxic at high concentrations and with prolonged cell exposure. We therefore titrated the CFSE labeling concentration and then assessed proinsulin-specific proliferative responses. PBMC were stained with varying concentrations of CFSE, then cultured with or without PI_33-63_, tetanus toxoid or anti-CD3 for 7 days (**Supplementary Figure 1**). The intensity of CFSE staining and the percentage of dead cells decreased as the CFSE labeling concentration decreased (**Figure 2**). A CFSE concentration of <0.025 µM was insufficient to detect dividing PI_33-63_, tetanus toxoid or anti-CD3–stimulated cells. A CFSE labeling concentration of 0.05 µM achieved the highest CDI and the best balance between CFSE staining intensity and PI_33-63_–specific proliferation (**Figure 2**).

### CFSE Staining Time

**Figure 3:**
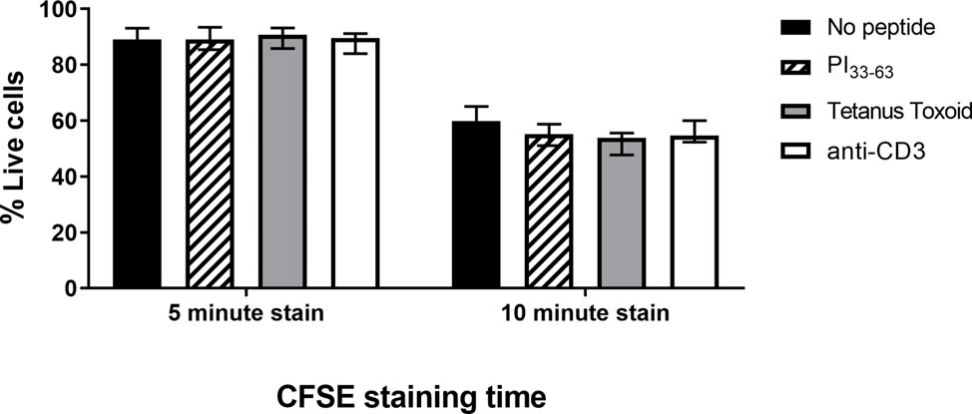
The influence of CFSE staining time on percentage of live cells after 7 days of incubation. Freshly isolated PBMC were stained with CFSE (0.05 µM) for 5 or 10 min and incubated with PI_33-63_ (10 µM), tetanus toxoid (10 Lf/ml), or anti-CD3 antibodies (0.1 µg/ml) for 7 days. Median value and range from three technical replicates are shown.

We next determined whether shortening CFSE staining duration would reduce toxicity. Freshly isolated PBMC were stained with 0.05 µM CFSE for 5 or 10 min before the CFSE reaction was terminated. PBMC were subsequently cultured with or without PI_33-63_, tetanus toxoid or anti-CD3 for 7 days (**Figure 3** and **Supplementary Figure 2**). A single analyst performed technical replicates on three patients with T1D ≤ 3 months. The percentage of live cells decreased, and intensity of CFSE staining and background proliferation increased with longer duration of CFSE staining. Staining for 5 min still resulted in sufficiently bright CFSE staining to discriminate divided and undivided cell populations (**Supplementary Figure 2**). CFSE staining time of 5 min, which resulted in the highest CDI, was selected to provide the best balance between cell death, background proliferation, and antigen-specific proliferation.

### Time Course of PBMC Proliferation

**Figure 4:**
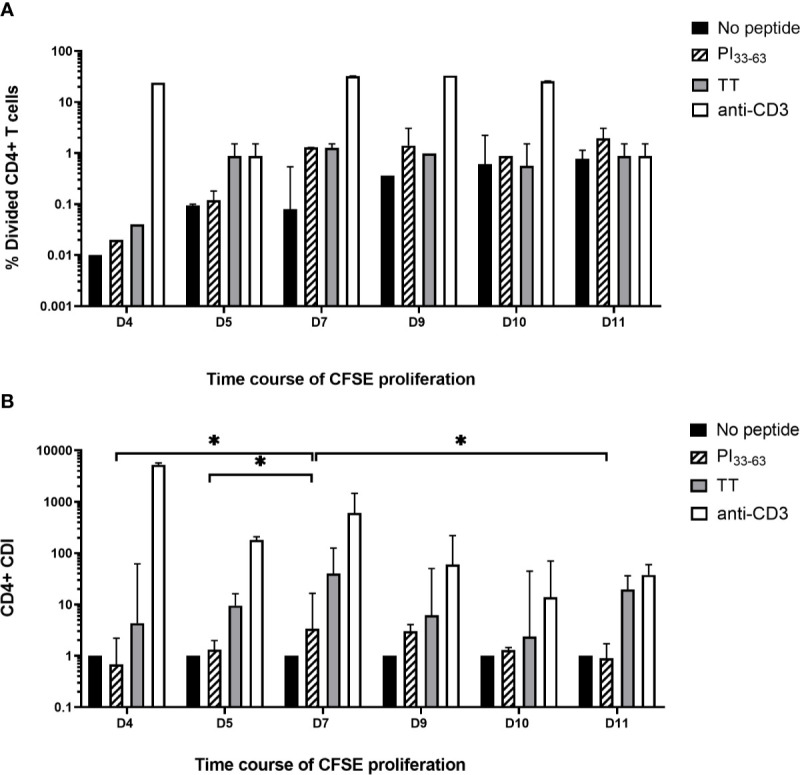
Time course of CFSE proliferation assay. Freshly isolated PBMC labeled with CFSE 0.05 uM for 5 min were cultured with no antigen, PI_33-63_, tetanus toxoid and anti-CD3 for 4, 7, 9, 10, or 11 days and the **(A)** percentage of divided cells and **(B)** CD4^+^ CDI were determined. The median and IQR CDI of 5 technical replicates is shown. *p < 0.05.

To determine the number of days in culture for optimal PI_33-63_–specific proliferation, CFSE-stained fresh PBMCs were cultured with medium alone, PI_33-63_, tetanus toxoid or anti-CD3 for 4, 5, 7, 9, 10, or 11 days. A single analyst performed technical replicates on five patients with T1D ≤ 3 months. With culture durations >7 days, antigen-specific proliferation did not change, but background proliferation in the absence of antigen increased, thus reducing the CDI (**Figure 4**). In cultures harvested earlier than 7 days, antigen-specific proliferation was reduced. The CDI was greatest at 7 days of culture (Day 4 vs. Day 7, p = 0.023; Day 5 vs. Day 7, p = 0.015; Day 7 vs. Day 9, ns; Day 7 vs. Day 10, p = 0.079; Day 7 vs. Day 11, p = 0.011). Day 7 was therefore selected in subsequent experiments as the optimal time to assess antigen specific proliferation.

### Comparison of Freshly Isolated and Frozen PBMC in the CFSE-Based T-Cell Proliferation Assay

**Figure 5:**
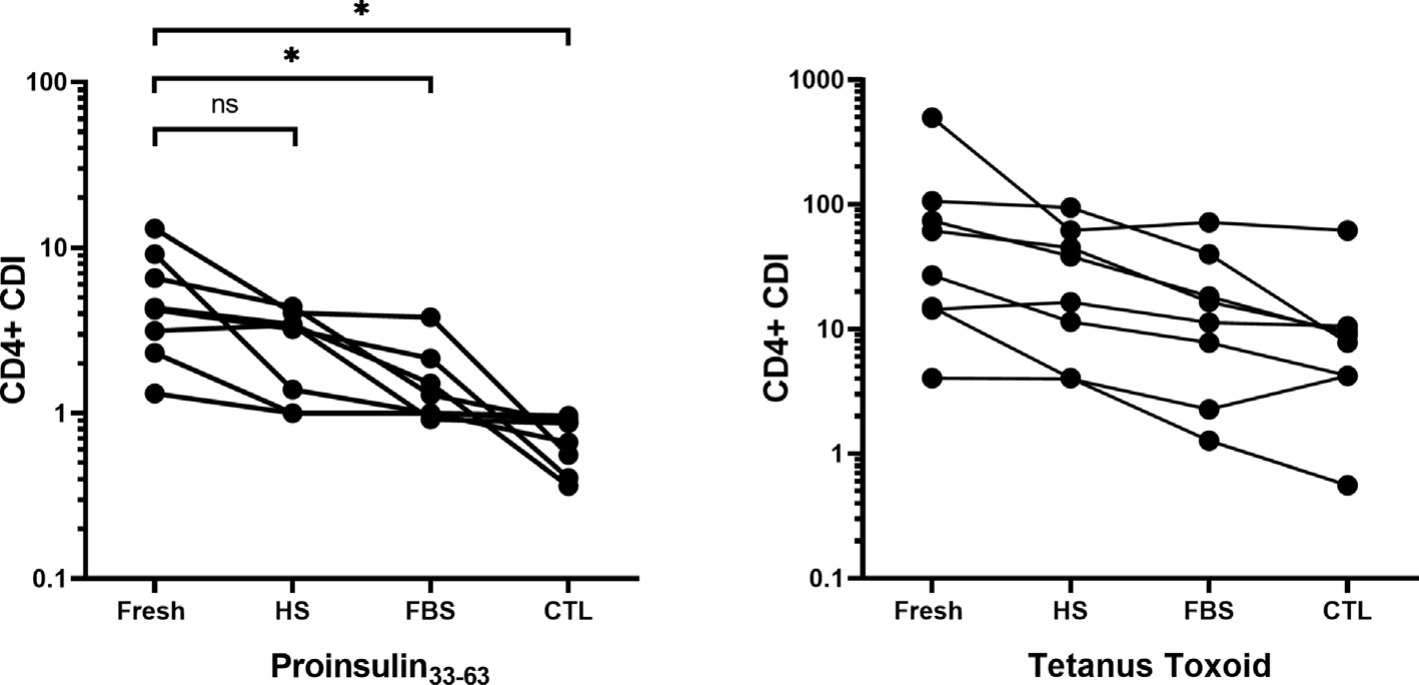
Comparison of the CD4^+^ T-cell division index using freshly isolated PBMCs cryopreserved in FBS/10% DMSO, HS/10% DMSO or serum-free CTL media, measured with the CFSE-based T-cell proliferation assay for eight different patients. Seven days after cryopreservation, frozen PBMCs were thawed and incubated without peptide, or with PI_33-63_, tetanus toxoid or anti-CD3 antibody. CDI for each freezing media was compared to CDI for fresh PBMCs: *p < 0.05 paired t test. Fresh, Freshly isolated PBMC; HS, Human Serum; FBS, Fetal Bovine Serum, CTL, CTL Cryo™ solution ABC.

T-cell proliferation assays would ideally measure responses in longitudinal studies or clinical trial samples in which frozen specimens from one patient would be compared at all time points in one run. CD4^+^ T responses to PI_33-63_, tetanus toxoid and human anti-CD3 were compared after cryopreservation in commonly used freezing media supplemented with Fetal Bovine Serum (FBS), Human Serum (HS), or *serum-free media (*CTL Cryo™ solution ABC) in eight separate patients (**Figure 5**). Five patients had T1D ≤ 3 months and three patients had T1D > 3 months. There was a significant decrease in CD4^+^ T-cell proliferative responses when cells were frozen in FBS and CTL Cryo™ solution compared to that detected with freshly isolated PBMC (*p = 0.016 and 0.014*, *respectively)*. PBMCs cryopreserved in HS/10% DMSO produced the lowest number of background events (**Supplementary Figure 3**) of all freezing media tested, and no sample was excluded based on positive control CDI ≤ 3. Based on tetanus toxoid-specific CDI ≤ 3 using PBMCs cryopreserved with CTL Cryo™ solution or FBS, one or two samples respectively would have been excluded. We conclude that CDI of PI_33-63_–specific assays conducted after freezing cells in HS/10% DMSO and thaw did not differ from CDI for the same assays conducted fresh (**Figure 5**).

### VPD450-Based T-Cell Proliferation Assay

#### Dose Titration

**Figure 6:**
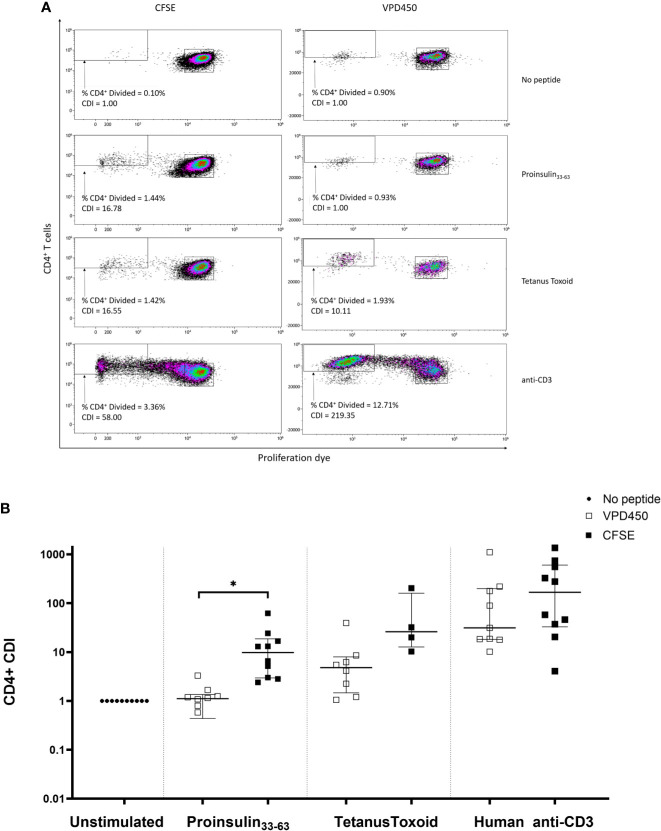
Comparison of CFSE-based T-cell proliferation assay (concentration 0.05µM, staining time 5 min) and VPD450 assay (concentration 0.25mM, staining time 15 min) in freshly isolated PBMCs from subjects with T1D. **(A)** A representative patient in whom CDI was determined for CD4^+^ T-cells in response to no antigen, PI_33-63_, tetanus toxoid, and anti-CD3. **(B)** Combined data from 10 patients, each tested with VPD450 and CFSE-based T-cell proliferation assays. Each point represents a CDI from an individual patient. CDIs for no antigen, PI_33-63_ and anti-CD3 are shown for all patients. CDIs for TT were measured for 10/10 and 4/10 patients using the VPD450 and CFSE-based T-cell proliferation assay, respectively. Error bars display median ± interquartile range. CDI for each CFSE-stained assay was compared to CDI for the corresponding VPD450-stained assay: *p < 0.05 paired t test.

As CFSE was cytotoxic at high labeling concentrations or with prolonged labeling, we compared CFSE with an alternative membrane dye, VPD450 (19). Freshly isolated PBMCs were labeled with varying concentrations of VPD450 for 15 min as recommended by the manufacturer and cultured for 7 days. At a VPD450 concentration of 0.1 µM, background proliferation of the no-peptide control was much higher than for the CFSE no-peptide control, and similar to the PI_33-63_–specific response (**Figure 6A**). VPD450 > 0.5 µM increased labeling intensity but reduced both % live cells and % divided cells after culture (**Supplementary Figure 4**). Tetanus Toxoid-specific CD4^+^ T-cell proliferation was maximal at 0.25 µM VPD450 and this concentration was selected to provide the best balance between staining intensity and CDI.

#### Comparison of CFSE-Based or VPD450-Based Proliferation Assay for CD4^+^ T-Cell Proliferation

T-cell responses to PI_33-63_, tetanus toxoid and anti-CD3 were compared using a CFSE-based or VPD450-based T-cell proliferation assay in freshly isolated PBMCs obtained from each of 10 patients with T1D of ≤ 3 months duration. Notably, VPD450 increased background proliferation in the absence of antigen, which reduced the antigen-specific CDI (**Figure 6B**). No samples were excluded based on positive control CDI ≤3. PI_33-63_–specific proliferative responses detected with CFSE were of a significantly greater magnitude than those with VPD (*p = 0.0412)*.

### Sample Storage Time Prior to Processing

We determined whether the time from venepuncture to isolation of PBMCs from freshly isolated blood affected CDI in 3 patients with T1D ≤ 3 months. Each patient’s sample was processed at three different timepoints; within 4 h, 12–16 h, or 22–24 h after collection (**Supplementary Figure 5**). Samples were stored at room temperature on a benchtop shaker. Cell viability decreased and CD4^+^ T-cell proliferation in no-peptide controls increased with longer storage time, resulting in a reduction in antigen-specific CDI. A blood storage time of 4 h or less optimized viability of CDI.

### Intra-Assay Repeatability and Inter-Assay Reproducibility

#### CD4^+^ T-Cell Proliferation

**Figure 7:**
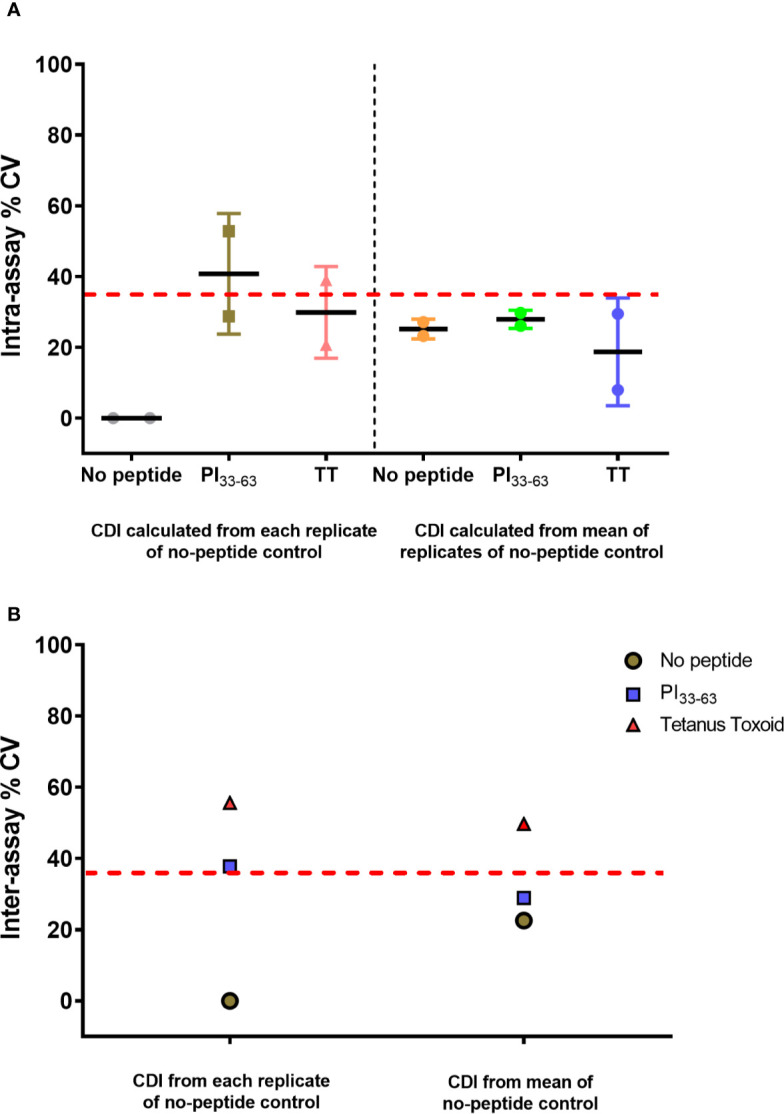
CD4^+^ CDI to PI_33-63_ and tetanus toxoid using cryopreserved PBMCs from a single patient with T1D ≤ 3 months. Assays were performed independently by two analysts. The mean CDI of triplicate measurements was calculated and the % Coefficient of Variation (CV) is shown. Each data point represents % CV. The solid lines represent mean % CV (intra-assay repeatability). The dashed red lines indicate the recommended threshold limit of CV ≤ 35% (18). **(A)** Intra-assay repeatability for CD4^+^ T-cell proliferative responses within assays performed by two independent analysts. **(B)** Inter-assay reproducibility for CD4^+^ T-cell proliferative responses between assays performed by two independent analysts.

To assess reproducibility, we produced a SOP for the assay and a qualification plan, and measured CD4^+^ T-cell responses to PI_33-63_ and tetanus toxoid from cryopreserved PBMC of a single patient diagnosed with T1D ≤ 3 months. Two analysts conducted independent experiments from the same sample. In each assay, six wells were set up for each condition. After culture with or without antigens, two wells were pooled together and analyzed as a single sample, resulting in three replicates. CDI were calculated from each replicate, or using mean value of replicates, of no-peptide wells. The intra-assay precision % CV was 52.82% for PI_33-63_–stimulated cells when CDI was calculated from each individual replicate of unstimulated cells. We found that small differences in the numbers of proliferating cells within the no-peptide control wells introduced large variations in CDI values. To minimize this variability, we used the mean value of no-peptide control triplicates to calculate antigen-specific CDI. This improved the overall precision outcome, including precision for PI_33-63_–stimulated cells to 29.73% CV, to within an acceptable level (**Figure 7**). The intra-assay repeatability was also within the recommended limit of CV ≤ 35% (18) when CDI values were calculated using the mean value of no-peptide control triplicates. Inter-assay reproducibility was within recommended limits for PI_33-63_ (18), but not for tetanus toxoid when calculating CDI values using the mean of replicates of no-peptide controls. Thus, it is recommended 1) that conditions are assayed in triplicate, preferably first pooling wells, to determine the mean value of no-peptide control replicates, and 2) that for time-point comparisons, one analyst runs all samples from a donor simultaneously in one assay.

**Figure 8:**
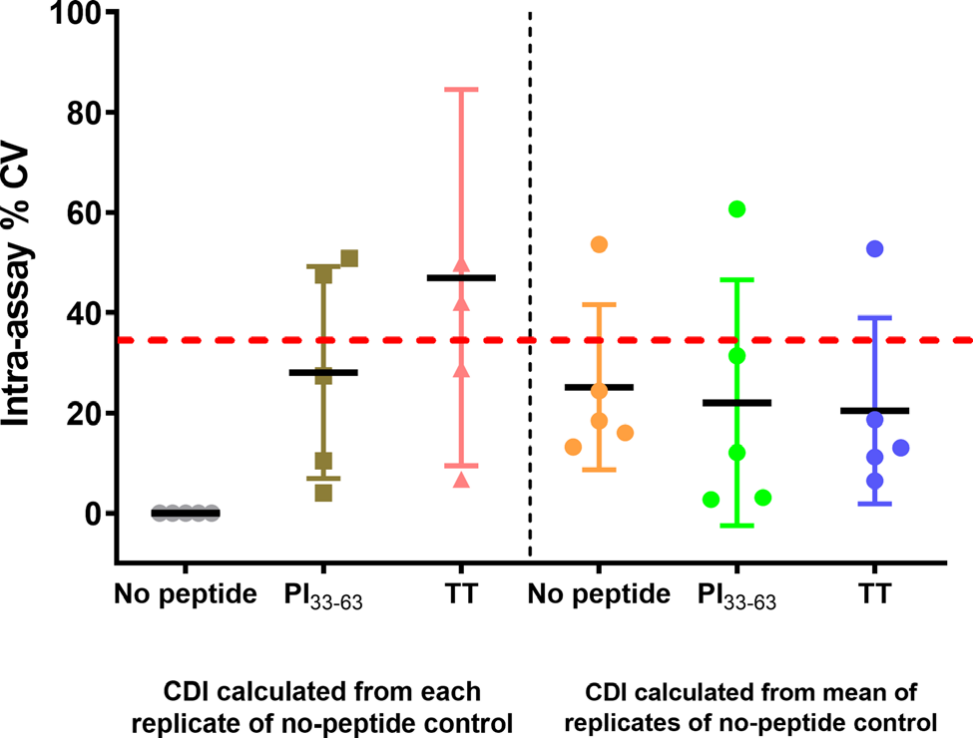
CD4^+^ CDI to PI_33-63_ and TT using freshly isolated PBMC from five patients with T1D ≤ 3 months, measured by a single analyst. From the mean CDI of triplicate measurements, the % Coefficient of Variation (CV) was calculated. Each data point represents % CV. Solid lines represent mean % CV (intra-assay repeatability). The dashed red line indicates the recommended threshold limit of CV ≤ 35% (18).

To assess precision in a larger group of patients, one analyst measured CD4^+^ T-cell responses to PI_33-63_ and tetanus toxoid from freshly isolated PBMCs of five patients diagnosed with T1D ≤ 3 months (**Figure 8**). Intra-assay precision was within the recommended limit of CV ≤ 35% (18) for PI_33-63_–stimulated cells. When CDI values were calculated using the mean value of replicates of no-peptide controls, the intra-assay precision for tetanus toxoid-stimulated cells was within an acceptable level.

## Discussion

T-cell assays can be used as biomarkers to monitor immune responses that are crucial to validate the outcome of clinical studies. Such assays can be incorporated into biomarkers to define disease heterogeneity and to monitor the rate of disease progression. In order for T-cell biomarkers to achieve broad utility, the assays by which they are measured must shift from requiring specialized expertise into optimized and validated assays for widespread clinical application. The CFSE-based T-cell proliferation assay is a promising technique to measure antigen-specific T-cell proliferation (10). However, there are currently no guidelines standardizing CFSE-based antigen specific T-cell proliferation assays. Here, we addressed key assay parameters for detection of autoantigen PI_33-63_–specific CD4^+^T-cell proliferation in T1D. We demonstrate the importance of using objective gating parameters, selecting the ideal dye and its concentration, labeling time, the optimal harvest time of PBMCs after culture, storage time before PBMCs are processed and cryopreservation media.

Optimizing a fluorescent dye-based proliferative assay involves balancing between staining PBMCs as brightly and homogenously as possible, while maintaining cell viability and minimizing background fluorescence from no-peptide controls. Higher concentrations of CFSE increased signal intensity but were associated with higher cell toxicity. While higher CFSE concentrations decreased PI_33-63_–specific CD4^+^ T-cell proliferation, reducing CFSE incubation times reduced background. Furthermore, we observed that different CFSE batches varied in staining intensity, impacting cell viability and proliferation in response to antigenic stimulation. Thus, CFSE titration studies should determine optimal concentration and staining time for labeling intensity and cell viability for each new batch of CFSE. For this reason, the current study employed the same batch of CFSE for all experiments in this paper. Other factors that minimized background proliferation and thereby increased the sensitivity of the assay included filtering human serum and meticulous attention to sterile technique. We demonstrated that minimizing the sample handling time prior to assay improves cell viability and antigen-specific proliferation. The sample storage period prior to processing reflected a time interval that can be feasibly implemented within a clinical research setting. We recognize that transit time, along with other factors such as changes in temperature, may affect cell viability and function and should be evaluated prior to implementation under other conditions.

Antigen-specific proliferation with the CFSE-based T-cell proliferation assay has mostly been tested using freshly isolated PBMCs. However, assays that use cryopreserved PBMCs are critical to investigate longitudinal changes in T-cell activity retrospectively. We observed that the cryopreservation medium influenced antigen-specific responses but that the CDIs obtained from PBMCs cryopreserved in HS were comparable to those of freshly isolated PBMCs. This was largely due to lower proliferation in the no-peptide control when using HS cryopreservation as compared to FBS cryopreservation where low-level proliferation could be induced by bovine-derived antigens present in FBS. Since cytokines and growth factors found in serum may influence lymphocyte responses (20), the use of a pooled large serum batch is preferable. Although previous studies suggested serum-free media reduces background events in no-peptide control wells in ELISPOT (20, 21), no such advantage was observed for the CFSE-based T-cell proliferation assay.

The capacity of cell permeant dyes such as CFSE to label lymphocyte populations with a high fluorescent intensity offers several advantages over ELISPOT. Proliferation assays enable more specific analysis of cell proliferation over several divisions. In addition, dividing cells can be characterized phenotypically using antibodies specific for surface markers and/or intracellular cytokines, which increases the information that can be obtained from a single functional assay. Since CFSE is a fluorescein-based dye it is also compatible with a broad range of other fluorochromes making it applicable to multi-color flow cytometry. In the case of ELISPOT assays, non-proliferating T cells or non-T cells may also produce cytokines, thus potentially reducing specificity of the assay to detect cytokine production from antigen-reactive T cells. CFSE also permits cloning of antigen-reactive cells *via* single-cell sorting. Thus, the scope and potential of the CFSE proliferation assay may exceed the output of the ELISPOT assay. When optimized, the CFSE proliferative assay is a sensitive means to detect antigen-specific proliferation.

To date, few comparative studies using alternative labeling dyes to CFSE have been conducted. We found that an assay based on CFSE was more sensitive in detecting antigen specific CD4^+^ T-cell proliferative responses using freshly isolated PBMCs than a VPD-450–based assay, due to higher background proliferation. While CFSE was superior toVPD450 in detecting PI_33-63_–specific CD4^+^ T-cell proliferation using our assay protocol, we did not attempt to further optimize parameters for VPD450, nor did we run trials with any other dye, such as cell trace violet (CTV). It is possible that further optimization of the VPD450-based T-cell proliferation assay, such as a reduction in labeling time, may have improved assay outputs. Alternatively, it is possible that issues with spectral overlap, whereby non-specific signal cannot be removed within the live/dead stain, resulted in a higher perceived VPD450 dilution in the unstimulated samples.

The reproducibility of T-cell assays is dependent on the optimization and transferability of the assays that are used. The Immune tolerance Network and Type 1 Diabetes TrialNet have tested various antigen-specific T-cell based assays and have shown the highest sensitivity and specificity for an immunoblot assay and ELISpot measurements. However, these assays are yet to overcome the effects of sample preparation (e.g., freshly isolated PBMC versus frozen biosamples). Furthermore, immune response assays using pooled peptides for T cell stimulation may be unsuitable for measurement of antigen-specific T cell responses in clinical trials of antigen-specific immunotherapy (22). Attempts to validate the detection of antigen-specific CD4^+^ T-cells with HLA class 1 multimers and class II tetramers demonstrated good reproducibility in individual laboratories but variable results across different laboratories (23, 24). As such, there are few widely usable, optimized assays for antigen-specific biomarkers in T1D.

We highlight the importance of the following critical parameters for reproducibility: recovery and viability of thawed PBMC, CFSE staining concentration and duration, standardized gating of divided cells, and pooled wells to improve numbers captured for flow cytometry analysis. Despite this, occasional no-peptide control wells had high cell division “noise”. Therefore, running the assay in triplicate, and using mean of unstimulated proliferation to calculate CDI greatly improved intra-assay repeatability for the CDI value. We also noted reduced inter-assay reproducibility for the very high CDIs obtained after tetanus toxoid stimulation. This may have resulted from small variations in the tetanus toxoid CFSE dilution profile between analysts, due to small differences in pipetting CFSE at assay set-up, which are amplified over a 7-day CFSE dilution assay. This variability can be overcome when a single analyst runs the assay, which would allow comparison of CDI over time or to different antigens in a clinical trial.

Here, we have systematically optimized and qualified a CFSE-based T-cell proliferation assay targeting rare antigen-specific CD4^+^ T-cells in children with T1D (10, 25). Toward the goal of establishing a fit-for-purpose assay, we have made every effort to comply with recognized standards of validation. The importance of strict attention to assay and analysis parameters has been highlighted, providing a protocol to allow harmonization across different laboratories. An advantage of this assay is the potential for the inclusion of phenotypic and functional determinations (26). Although it is known that T-cells are essential constituents of T1D disease progression, their phenotype is yet to be fully evaluated. Future efforts will be able to facilitate an integration of optimized flow cytometric approaches with newer technologies to create T-cell biomarkers that have single-cell resolution. For example, the CFSE-based proliferation assay can be paired with platforms such as multi-parameter cytometry (27) in order to phenotype an array of surface and intracellular markers. Transcriptomic and TCR analyses (28, 29) can incorporate single-cell approaches to shed light on pathogenic signatures or mechanisms underlying disease. Together these assays should enhance understanding of the immunogenic mechanisms underpinning T1D and the response to novel immune-modulatory agents.

## Data Availability Statement

The original contributions presented in the study are included in the article/**Supplementary Material**. Further inquiries can be directed to the corresponding author.

## Ethics Statement

The studies involving human participants were reviewed and approved by Children’s Health Queensland, Mater Hospital and University of Queensland Human Research Ethics Committees. Written informed consent to participate in this study was provided by the participants’ legal guardian/next of kin.

## Author Contributions

YM, SP, HN, EH-W, MH, and RT designed research. YM, HN, and NR performed research. YM, HN, EH-W, SP, MH, and RT analyzed data. YM, HN, NR, SP, EH-W, MH, and RT prepared the manuscript. All authors contributed to the article and approved the submitted version.

## Funding

This work was supported by a grant from The Leona M. and Harry B. Helmsley Charitable Trust and Juvenile Diabetes Research Foundation Australia to RT, MH, and EH-W. RT is supported by Arthritis Queensland and an NHMRC Research Fellowship. YM is supported by a University of Queensland Post-Graduate Award, Pfizer Australasian Paediatric Endocrine Care Grant, Butta Clinician Researcher Bursary, Children’s Health Foundation PhD Top up Scholarship, Royal Australasian College of Physicians Foundation Development and Research Entry Scholarship.

## Conflict of Interest

The authors declare that the research was conducted in the absence of any commercial or financial relationships that could be construed as a potential conflict of interest.
