# Supplementary material for: Optimization of a Method to Detect Autoantigen-Specific T-Cell Responses in Type 1 Diabetes
Source: Front Immunol. 2020 Dec 7;11:587469. doi: 10.3389/fimmu.2020.587469 (PMC7793893; doi:10.3389/fimmu.2020.587469)
Supplement: Supplementary file 1 [file DataSheet_1.pdf]

Supplementary Material

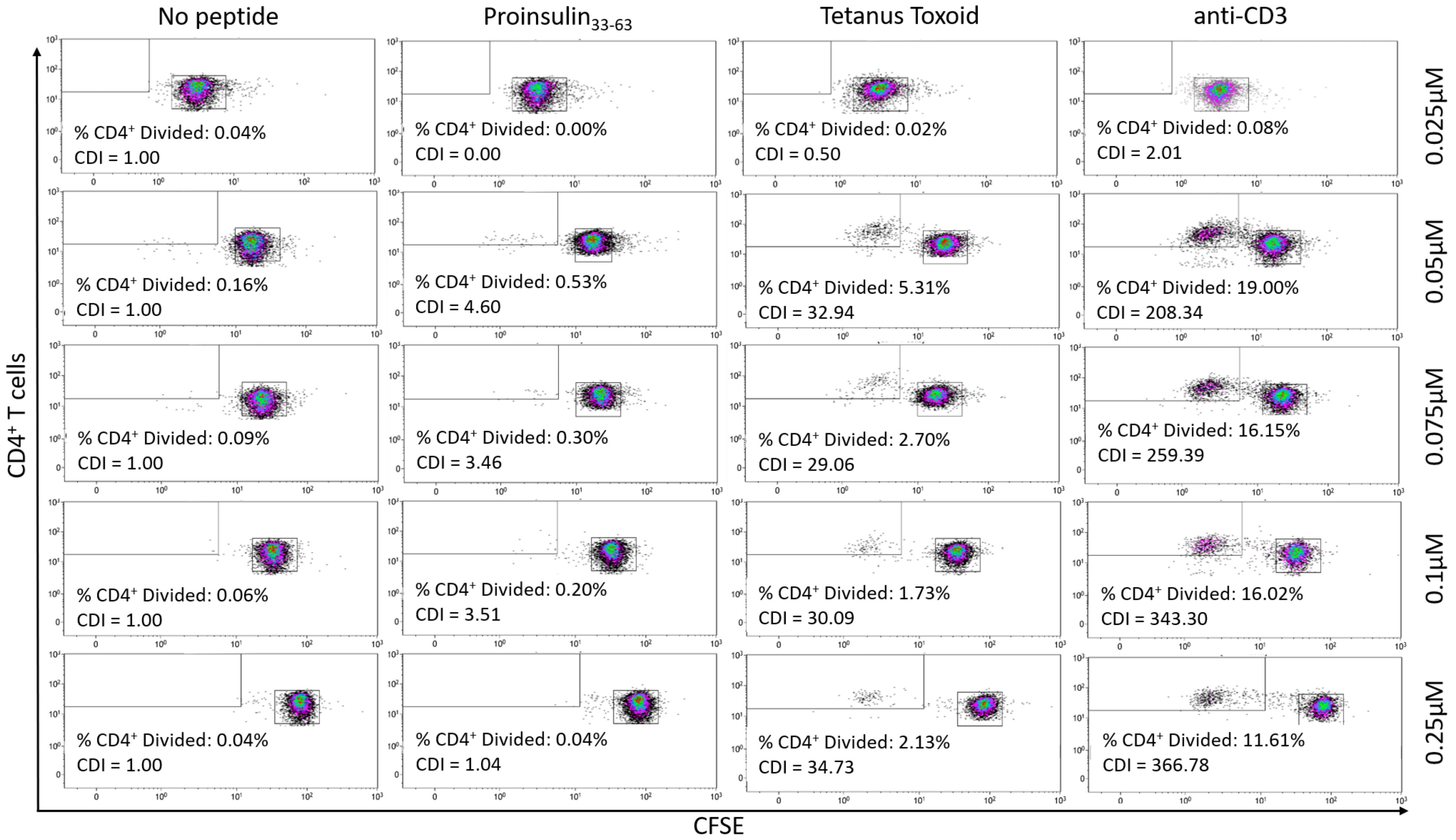

No peptide
Proinsulin33-63
Tetanus Toxoid
anti-CD3
% CD4+ Divided: 0.04%
CDI = 1.00
% CD4+ Divided: 0.00%
CDI = 0.00
% CD4+ Divided: 0.02%
CDI = 0.50
% CD4+ Divided: 0.08%
CDI = 2.01
0.025µM
% CD4+ Divided: 0.16%
CDI = 1.00
% CD4+ Divided: 0.53%
CDI = 4.60
% CD4+ Divided: 5.31%
CDI = 32.94
% CD4+ Divided: 19.00%
CDI = 208.34
0.05µM
% CD4+ Divided: 0.09%
CDI = 1.00
% CD4+ Divided: 0.30%
CDI = 3.46
% CD4+ Divided: 2.70%
CDI = 29.06
% CD4+ Divided: 16.15%
CDI = 259.39
0.075µM
% CD4+ Divided: 0.06%
CDI = 1.00
% CD4+ Divided: 0.20%
CDI = 3.51
% CD4+ Divided: 1.73%
CDI = 30.09
% CD4+ Divided: 16.02%
CDI = 343.30
0.1µM
% CD4+ Divided: 0.04%
CDI = 1.00
% CD4+ Divided: 0.04%
CDI = 1.04
% CD4+ Divided: 2.13%
CDI = 34.73
% CD4+ Divided: 11.61%
CDI = 366.78
0.25µM
CD4+ T cells
CFSE

Supplementary Figure 1. Titration of CFSE concentration. PBMC were stained with concentrations of CFSE as shown, then incubated with $PI_{33\text{-}63}$ (10µM), tetanus toxoid (10Lf/ml) or anti-human CD3 antibodies (0.1µg/ml) for 7 days. Cells were gated on live $CD4^+$ cells and at least 2 x 10^5 lymphocytes were acquired to identify 5000 $CD4^+$, $CFSE^{undivided}$ events (right hand gate). The number of $CD4^+$ $CFSE^{divided}$ events (left hand gate) was calculated. One of three independent experiments on paediatric patients with T1D $\leq$ 3 months is shown.

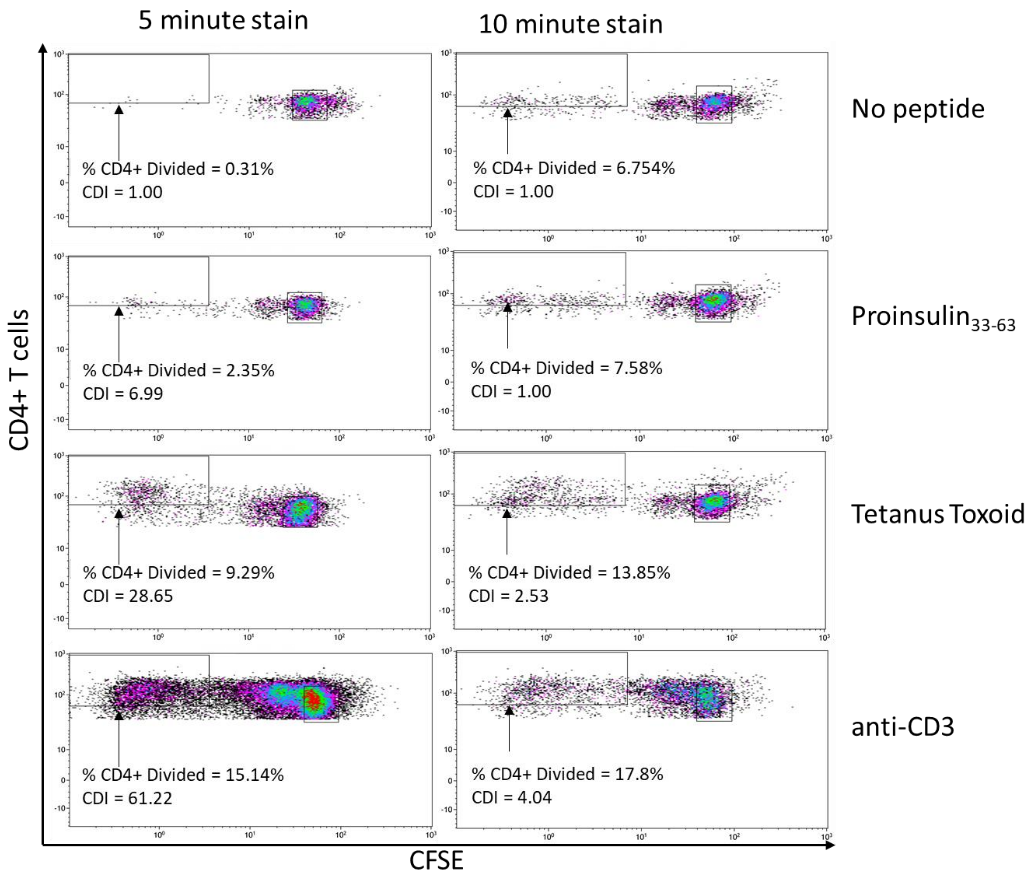

Supplementary Figure 2. Representative flow cytometry plots displaying the effect of varying CFSE staining time on no-peptide background proliferation. PBMCs were stained with CFSE (0.05µM) for 5 (left-hand panel) or 10 minutes (right-hand panel) and incubated with PI_{33-63} (10µM), tetanus toxoid (10Lf/ml) or anti-CD3 antibody (0.1µg/ml) for 7 days. One of three independent experiments is shown.

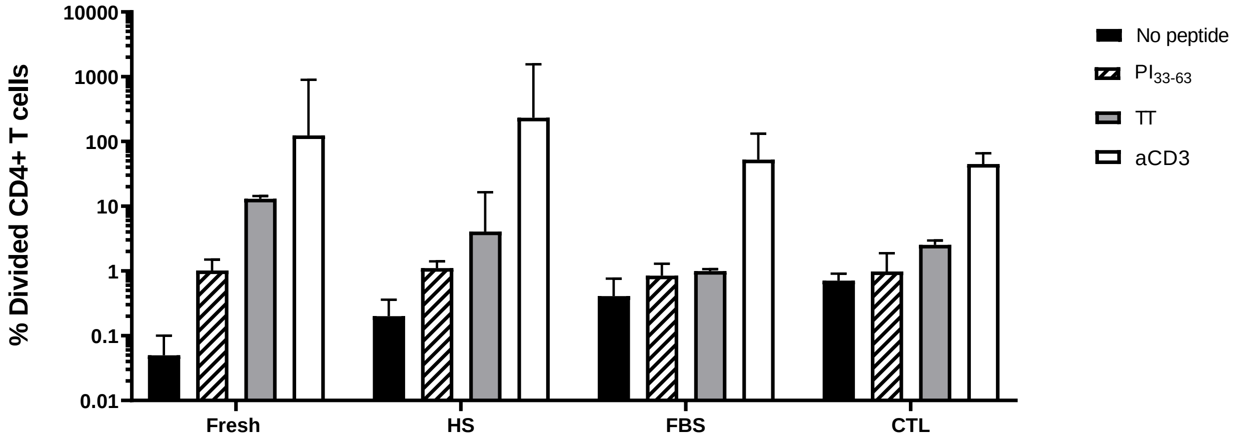

Supplementary Figure 3. Comparison of the percentage of Divided CD4^{+} T-cells using PBMCs cryopreserved in FBS/10% DMSO, HS/10% DMSO or serum-free CTL media, measured with the CFSE-based T-cell proliferation assay for three different patients. Seven days after cryopreservation, frozen PBMCs were thawed and incubated without peptide, or with $PI_{33\text{-}63}$, tetanus toxoid or anti-CD3 antibody. CDI for each freezing media was compared to CDI for fresh PBMCs:* $p<0.05$ paired t test. Median and range are displayed. Fresh, Fresh PBMC; HS, Human Serum; FBS, Fetal Bovine Serum, CTL, CTL Cryo™ solution ABC.

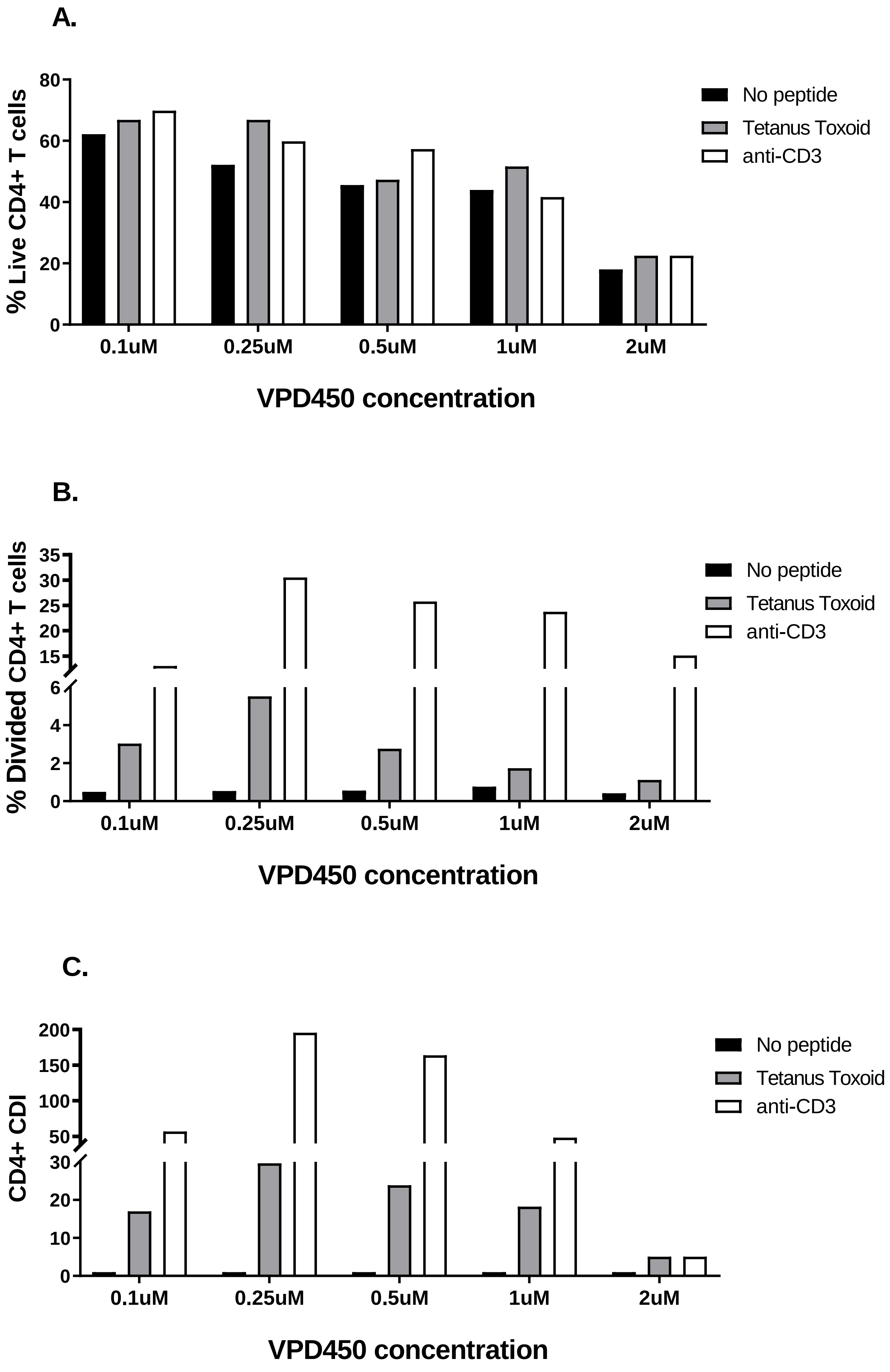
A.
% Live CD4+ T cells
80
60
40
20
0
0.1uM
0.25uM
0.5uM
1uM
2uM
VPD450 concentration
No peptide
Tetanus Toxoid
anti-CD3
B.
% Divided CD4+ T cells
35
30
25
20
15
6
4
2
0
0.1uM
0.25uM
0.5uM
1uM
2uM
VPD450 concentration
No peptide
Tetanus Toxoid
anti-CD3
C.
CD4+ CDI
200
150
100
50
30
20
10
0
0.1uM
0.25uM
0.5uM
1uM
2uM
VPD450 concentration
No peptide
Tetanus Toxoid
anti-CD3

Supplementary Figure 4. Titration of VPD450 dye concentration. PBMCs were stained with different concentrations of VPD450 for 15 minutes as shown and incubated with tetanus toxoid (10Lf/ml) or human anti-CD3 antibodies (0.1µg/ml) for 7 days. The cells were then stained with FITC anti-CD3 antibody and AF700 anti-CD4 antibody and analysed by flow cytometry after 7 days of culture at varying VPD450 concentrations. **(A)** Percentage of live cells. **(B)** Percentage of Divided $CD4^+$ T-cells. **(C)** $CD4^+$ Cell Division Index. One of three independent experiments is shown.

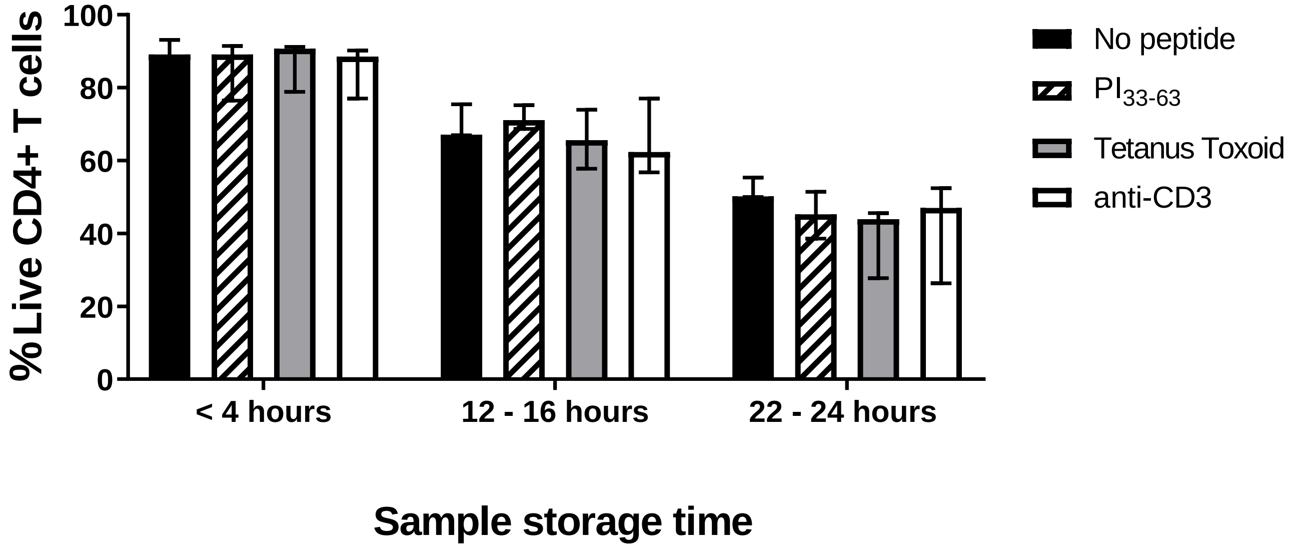

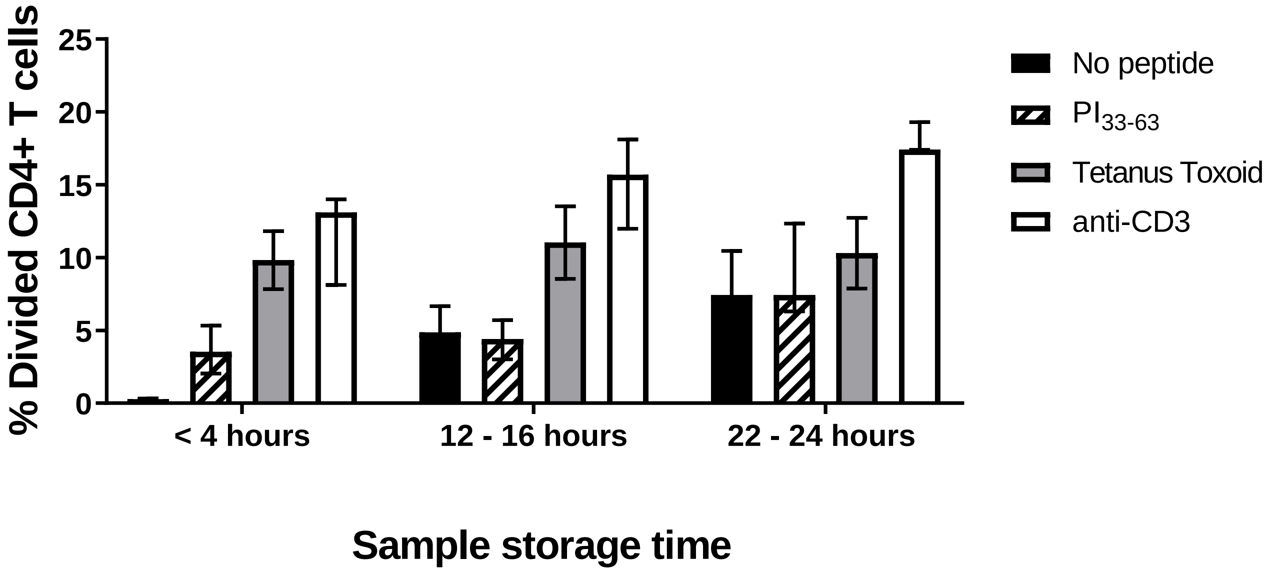

Supplementary Figure 5. The effect of sample storage time prior to processing on the survival and proliferation of PBMCs after 7 days of incubation. PBMCs were stained with CFSE (0.05µM) for 5 minutes and incubated with PI_{33-63} (10µM), tetanus toxoid (10Lf/ml) or anti-CD3 antibody (0.1µg/ml) for 7 days. Median value and range from 3-technical replicates are shown.
